# Development and validation of a nomogram model for predicting low muscle mass in patients undergoing hemodialysis

**DOI:** 10.1080/0886022X.2023.2231097

**Published:** 2023-07-05

**Authors:** Rongrong Tian, Liyang Chang, Ying Zhang, Hongmei Zhang

**Affiliations:** a Department of Blood Purification Centre, Hangzhou TCM Hospital Affiliated to Zhejiang Chinese Medical University, Hangzhou, Zhejiang, China; b Department of Science and Development, The Second Affiliated Hospital of Zhejiang University School of Medicine, Hangzhou, Zhejiang, China

**Keywords:** Muscle mass, hemodialysis, nomogram, handgrip strength, gait speed

## Abstract

**Background:**

Muscle mass is important in determining patients’ nutritional status. However, measurement of muscle mass requires special equipment that is inconvenient for clinical use. We aimed to develop and validate a nomogram model for predicting low muscle mass in patients undergoing hemodialysis (HD).

**Methods:**

A total of 346 patients undergoing HD were enrolled and randomly divided into a 70% training set and a 30% validation set. The training set was used to develop the nomogram model, and the validation set was used to validate the developed model. The performance of the nomogram was assessed using the receiver operating characteristic (ROC) curve, a calibration curve, and the Hosmer–Lemeshow test. A decision curve analysis (DCA) was used to evaluate the clinical practicality of the nomogram model.

**Results:**

Age, sex, body mass index (BMI), handgrip strength (HGS), and gait speed (GS) were included in the nomogram for predicting low skeletal muscle mass index (LSMI). The diagnostic nomogram model exhibited good discrimination with an area under the ROC curve (AUC) of 0.906 (95% CI, 0.862–0.940) in the training set and 0.917 (95% CI, 0.846–0.962) in the validation set. The calibration analysis also showed excellent results. The nomogram demonstrated a high net benefit in the clinical decision curve for both sets.

**Conclusions:**

The prediction model included age, sex, BMI, HGS, and GS, and it can successfully predict the presence of LSMI in patients undergoing HD. This nomogram provides an accurate visual tool for medical staff for prediction, early intervention, and graded management.

## Background

Sarcopenia is highly prevalent in patients undergoing hemodialysis (HD) and is associated with adverse clinical outcomes [1–3]. Therefore, it is crucial to identify sarcopenia in a timely manner for early intervention. Muscle mass is a key component of sarcopenia. However, the measurement of muscle mass requires special equipment such as dual-energy X-ray absorptiometry (DEXA), magnetic resonance imaging (MRI), and bioelectrical impedance analysis (BIA) [4]. The equipment has some disadvantages, such as high cost and poor accessibility, which limit its wide clinical application [5–7]. Very few dialysis centers have direct access to these devices. Consequently, sarcopenia is often underdiagnosed and undertreated in clinical practice. In addition to sarcopenia, muscle mass measurements are included in other approaches used to define nutritional status, such as protein energy wasting [8], malnutrition [9], and cachexia [10]. Therefore, developing and validating an optimal tool that can estimate muscle mass based on simple parameters is of paramount clinical relevance.

The Global Leadership Initiative on Malnutrition (GLIM) recently updated the definition of malnutrition in 2019 [9]. A reduction in muscle mass is a diagnostic criterion. Considering that muscle mass measurement using specialized equipment is still not available in most institutions worldwide, the GLIM consensus states that assessment of muscle function, such as handgrip strength (HGS), can be used as a supportive measure for muscle mass [9]. The prognostic value of malnutrition, defined according to the GLIM criteria using HGS, has been verified in cancer patients [11]. Very recently, it was reported that gait speed (GS) is another predictor of low muscle mass in cancer patients, and a model for low muscle mass prediction including GS was developed and validated [12]. Among patients on HD, serum creatinine, a routinely measured biochemical marker, was also considered to be correlated with muscle mass [13]. Assessing these factors is generally advantageous because of their simplicity, low cost, and ease of use [14,15]. However, the value of these nutritional status markers such as HGS and GS, assessed alone or in combination with serum creatinine, for predicting low muscle mass has rarely been investigated in patients undergoing HD, although the utility of HGS in estimating lean body mass has been investigated in chronic kidney disease (CKD) patients not receiving dialysis [16].

Nomograms have recently received increasing attention and have been widely used to predict the risk of disease. A nomogram is a picture that is used to visualize the data analysis results. The form of the prediction tool is visual and intuitive [17]. It has been shown to enable more accurate prediction for individual patients with diverse types of disease [18–21]. Recent studies have developed and validated a nomogram for predicting sarcopenia in community-dwelling older adults and low muscle mass in patients with gastric cancer [12,17]. Those models can facilitate early identification and timely intervention for high-risk populations. Therefore, our study aimed to develop and validate a nomogram model using conveniently measured factors for the early identification of a low skeletal muscle mass index (LSMI), thus facilitating timely diagnosis and treatment in clinical practice.

## Methods

### Subjects and study design

We included all patients who participated in the previous cross-sectional study from September 2020 to January 2021 in our hemodialysis center [22]. The inclusion criteria included being at least 18 years old and being on maintenance hemodialysis three times weekly for ≥ eight weeks. The exclusion criteria included the following: (1) patients who could not undergo BIA, (i.e., those who underwent pacemaker installation), or patients who underwent amputation surgery; (2) those with an acute infection; (3) those who had cardiovascular events or hospitalization within three months; (4) those who had been diagnosed with malignancies; (5) those with muscular and neuromuscular disorders; (6) those with severe edema or cognitive dysfunction; and (7) those confined to bed or a wheelchair. All patients provided written informed consent to participate in the study. The ethics committee of the hospital approved the study (no. 2020KY116).

### Clinical, laboratory and nutritional parameters

Collection and measurement of samples were performed as previously described [22]. Age, sex, dialysis vintage, primary kidney disease, body mass index (BMI), residual kidney function (RKF), and laboratory data were collected at the time of research recruitment. RKF was defined as a 24-h urine output of more than 200 mL. All laboratory parameters, including hemoglobin and serum concentrations of creatinine [SCr], blood urea nitrogen [BUN], hypersensitive C-reactive protein [hs-CRP], triglyceride [TG], total cholesterol [TCH], serum phosphorus, serum albumin, and total protein, were tested using fasting blood samples collected before dialysis at the midweek session. Intact parathyroid hormone [iPTH] assays were used to determine serum parathyroid hormone concentrations. Single-pool Kt/V for urea was calculated as an indicator of dialysis dose, and normalized protein equivalent of nitrogen appearance (nPNA) was calculated as an indicator of protein metabolism [23].

The Modified Quantitative Subjective Global Assessment (MQSGA) was used as an indicator of nutritional status. There are seven variables with a total score ranging from 7 to 35; a score of 7 indicates normal and 35 indicates severe malnutrition [24]. It has been widely used in the assessment of nutritional status in hemodialysis patients [25,26].

### Measurement of muscle function

Muscle function was measured using HGS or GS [27]. HGS was measured using a grip strength dynamometer (Guangdong Xiangshan Weighing Apparatus Group, China) before dialysis. All patients stood with their elbows fully extended. They were asked to squeeze the dynamometer using the non-fistulated hand (or the dominant hand for patients with central venous catheters). HGS was assessed in two consecutive trials with a 1-min interval between each trial, and the highest reading was recorded for analysis. GS was evaluated by a 6-m walking test at a normal pace before dialysis. A 10-m walking line was prepared with separate sections for walking and measurement. Participants were instructed to walk the distance at their normal pace, while the time taken to cover the 6-m measurement section was recorded using a stopwatch. The average result of the two trials was calculated and recorded.

### Assessment of muscle mass

Skeletal muscle mass was measured by a multifrequency BIA device, model Seca515 (Seca GmbH & Co., Germany). To eliminate the influence of excessive body fluids on the measurement, BIA tests were timed after HD treatment, when patients reached the estimated dry weight. The estimated dry weight was determined by a nephrologist using clinical features, blood pressure, chest radiograph, inferior vena cava diameter, and serum natriuretic peptide level. According to the Kidney Disease Outcomes Quality Initiative (KDOQI) clinical practice guideline for BIA use in adults undergoing HD, BIA measurements were performed 30 min after the end of the dialysis session to allow for redistribution of body fluids [28]. All patients were asked to eat only small snacks (e.g., small biscuits) during HD to prevent hypoglycemia and to fast for more than 2 h before the BIA measurement. The skeletal muscle index (SMI) was computed by dividing the skeletal muscle mass (kg) by the square of the height (m) [29]. LSMI was defined as < 7.0 kg/m^2^ for males and < 5.7 kg/m^2^ for females [27].

### Physical activity level measurements

The international physical activity questionnaire (IPAQ) was used to assess physical activity level (PAL). It has been tested and has demonstrated acceptable reliability and validity [30]. The questionnaire mainly evaluated the occupation, housework, transportation, and leisure physical activity of patients during the last seven days.

### Statistical analysis

The study dataset was randomly divided into a 70% training set and a 30% validation set. The training set was used to develop the nomogram model, and the validation set was used to validate the developed model. Normally and nonnormally distributed continuous variables are presented as the mean ± standard deviation (SD), median, and interquartile range. Categorical variables were described as numbers (percentages). Comparisons of the groups were conducted using Student’s *t* test, the Mann–Whitney U test, or the chi-square test, depending on the type of variables.

Logistic regression analysis was used to construct a prediction model for LSMI in the training set. Variables with statistical significance in the univariate analysis (*p* < .05) were included in the multivariate logistic regression analysis through a backward conditional process. Based on the independent predictive factors, a nomogram was developed. Every variable in the nomogram plot could obtain a corresponding score on the top point line (0–100 points by default), and the final score was equal to the sum of the scores for each variable [17]. This equation was also used to calculate the total points for each patient. The total points were then incorporated into the receiver operating characteristic (ROC) curve to obtain the optimal cutoff value. The discrimination performance of the nomogram model for predicting LSMI was analyzed using ROC curves in both the training and validation sets. In general, an area under the ROC curve (AUC) of 0.5 indicates no discrimination, 0.7 to 0.8 is considered acceptable, 0.8 to 0.9 is considered excellent, and more than 0.9 is considered outstanding [31]. A *p* value >.05 in the Hosmer–Lemeshow test indicates a high calibration performance. Decision curve analysis (DCA) was used to evaluate the clinical practicality of the nomogram model.

Statistical analyses were performed using SPSS (version 23.0; IBM, Armonk, NY) and R software, version 4.1.3 (R Project for Statistical Computing, Vienna, Austria). All statistical tests were two-sided, and a *p* value <.05 was regarded as statistically significant.

## Results

### Baseline characteristics of the study population

**Figure 1.**
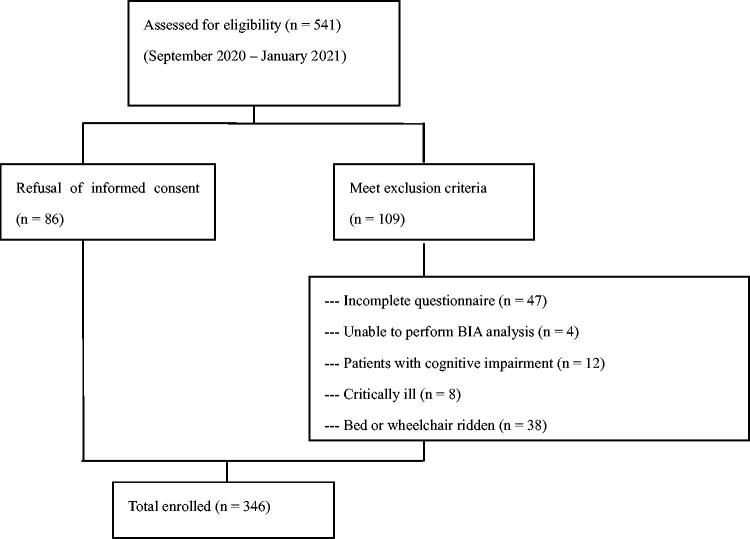
Flow chart of the participants. *Note*: ‘critically ill’ refers to individuals who had experienced cardiovascular events or hospitalization within the past 3 months, or who had acute infections or malignancies.

In total, 541 patients were assessed for eligibility between September 2020 and January 2021. We excluded 109 patients who met the exclusion criteria and 86 patients who refused to provide informed consent. A flowchart representing the patient selection process is presented in Figure 1. Finally, 346 patients were enrolled and randomly divided into a training cohort (*n* = 244) or a validation cohort (*n* = 102).

Of the 346 patients on maintenance hemodialysis (MHD), 129 were diagnosed with LSMI, with a prevalence of 37.3%. Patients with low muscle mass were significantly older than those with normal muscle mass. SCr, albumin, phosphorus, SMI, BMI, HGS, and GS were lower, whereas MQSGA were higher in the low muscle mass group. The baseline characteristics of the training and validation sets are presented in Table 1.

**Table 1.** Baseline characteristics of the training set and validation set.

	Training set (*n* = 244)			Validation set (*n* = 102)		
	Low SMI (*n* = 84)	Normal (*n* = 160)	*p*	Low SMI (*n* = 45)	Normal (*n* = 57)	*p*
Age (years)	66.81 ± 12.95	54.13 ± 12.22	<.001	66.02 ± 9.18	50.60 ± 12.74	<.001
Sex						
Male, n (%)	42 (50.0%)	47 (29.4%)	.001	24 (53.3%)	20 (35.1%)	.065
Dialysis vintage, months	40(22, 98)	45 (16, 98)	.622	64(23.50, 120.50)	60(30, 107)	.764
ESRD primary cause			.896			.247
Diabetic nephropathy, n(%)	24(28.6%)	47(29.4%)		14 (31.1%)	12 (21.1%)	
Others, n(%)	60(71.4%)	113(70.6%)		31 (68.9%)	45 (78.9%)	
RKF, n (%)	19(22.6%)	34(21.3%)	.805	8 (17.8%)	10 (17.5%)	.975
Kt/V for urea	1.69 ± 0.40	1.43 ± 0.34	<.001	1.67 ± 0.44	1.52 ± 0.56	.157
BMI (kg/m^2^)	21.01 ± 2.66	23.83 ± 3.62	<.001	20.26 ± 2.35	23.32 ± 2.95	<.001
Biological parameters						
BUN (mmol/L)	20.55 ± 6.87	22.26 ± 5.26	.031	23.08 ± 9.39	22.93 ± 5.68	.920
SCr (μmol/L)	733.40 ± 199.39	930.19 ± 245.48	<.001	728.78 ± 182.17	954.47 ± 276.88	<.001
TCH (mmol/L)	4.35 ± 1.12	3.98 ± 0.93	.006	4.15 ± 0.96	4.14 ± 1.62	.950
TG (mmol/L)	1.43 (1.04, 1.98)	1.63 (1.03, 2.57)	.188	1.29 (0.92, 2.03)	1.44 (1.12, 2.26)	.139
Hs-CRP (g/L)	2.37 (1.06, 5.86)	1.95 (1.02, 4.77)	.308	2.83 (1.04, 5.42)	2.71 (1.21, 5.68)	.882
Albumin (g/L)	38.57 ± 3.21	39.51 ± 3.40	.038	38.38 ± 3.11	40.00 ± 2.73	.007
Total protein (g/L)	64.71 ± 5.25	65.52 ± 5.26	.256	64.54 ± 4.39	66.02 ± 5.25	.133
Hemoglobin (g/L)	108.62 ± 15.84	111.95 ± 14.97	.107	111.00 ± 15.76	114.37 ± 15.44	.281
iPTH (pg/mL)	272.60 (142.85, 449.58)	321.90 (193.68, 563.79)	.141	307.20 (77.90, 549.75)	355.20 (177.45, 653.00)	.173
Phosphorus (mmol/L)	1.62 ± 0.35	1.87 ± 0.43	<.001	1.74 ± 0.43	2.07 ± 0.73	.005
nPNA (g/kg/d)	1.10 ± 0.36	1.09 ± 0.24	.701	1.22 ± 0.48	1.13 ± 0.24	.310
MQSGA score	11.83 ± 2.48	10.28 ± 1.77	<.001	12.60 ± 2.56	10.37 ± 1.41	<.001
BIA						
SMI (kg/m^2^)	5.75 ± 0.99	8.00 ± 1.31	<.001	5.75 ± 0.95	7.88 ± 1.24	<.001
HGS (kg)	21.51 ± 6.65	31.03 ± 9.09	<.001	22.30 ± 8.06	31.46 ± 9.62	<.001
GS (m/s)	0.88 ± 0.19	1.15 ± 0.25	<.001	0.94 ± 0.18	1.26 ± 0.23	<.001
PAL (MET)	1040.92 ± 736.47	1294.50 ± 818.13	.018	1137.56 ± 837.89	1366.05 ± 845.19	.177

*Notes:* Values for continuous variables are given as the means ± standard deviations or medians and interquartile ranges. Categorical variables are expressed as numbers (%). RKF: residual kidney function; BMI: body mass index; BUN: blood urea nitrogen; SCr: serum creatinine; TCH: total cholesterol; TG: triglyceride; hs-CRP: high-sensitivity C-reactive protein; iPTH: intact parathyroid hormone; nPNA: normalized protein equivalent of nitrogen appearance; MQSGA: Modified Quantitative Subjective Global Assessment; SMI: skeletal muscle mass index; HGS: handgrip strength; GS: gait speed; PAL: physical activity level; MET: metabolic equivalent.

### Derivation of the predictive factors in the training set

Univariate analysis showed that age, sex, Kt/V, BUN, SCr, TCH, albumin, phosphorus, MQSGA, BMI, HGS, GS and PAL were associated with the LSMI in the training set (Table 2). As phosphorus-lowering drugs and cholesterol-lowering drugs are frequently prescribed to the HD population and have a significant impact on the predictive ability of serum phosphorus and TCH levels for low muscle mass, we excluded them in the multivariate analysis. In multivariate logistic regression analysis, age (1.090; 95% CI, 1.045–1.138), sex (0.239; 95% CI, 0.068–0.840), BMI (0.515; 95% CI, 0.415–0.641), HGS (0.851; 95% CI, 0.780–0.929), and GS (0.015; 95% CI, 0.001–0.206) were independently associated with LSMI (Table 3).

**Table 2.** Univariate analysis for LSMI in the training set.^a^

Variables	OR (95% CI)	*p*
Age (years)	1.087 [1.059, 1.115]	<.001
Sex (female)	0.416 [0.241, 0.718]	.002
Kt/V for urea	9.584 [3.640, 25.238]	<.001
BUN (mmol/L)	0.944 [0.896, 0.995]	.031
SCr (μmol/L)	0.996 [0.995, 0.997]	<.001
TCH (mmol/L)	1.444 [1.101, 1.894]	.008
Albumin (g/L)	0.920 [0.850, 0.997]	.041
Phosphorus (mmol/L)	0.195 [0.092, 0.416]	<.001
MQSGA score	1.433 [1.241, 1.655]	<.001
BMI (kg/m^2^)	0.731 [0.654, 0.816]	<.001
HGS (kg)	0.858 [0.820, 0.898]	<.001
GS (m/s)	0.002 [0.000, 0.014]	<.001
PAL (MET)	1.000 [0.999, 1.000]	.019

*Note:* LSMI: low skeletal muscle mass index; OR: odds ratio; CI: confidence interval; BUN: blood urea nitrogen; SCr: serum creatinine; TCH: total cholesterol; MQSGA: Modified Quantitative Subjective Global Assessment; BMI: body mass index; HGS: handgrip strength; GS: gait speed; PAL: physical activity level; MET: metabolic equivalent.

^a^Variables with *p* value <.05 in the univariate analysis are presented in the table.

**Table 3.** Multivariate logistic regression analysis for LSMI in the training set.^a^

Variables	OR (95% CI)	*p*
Age (years)	1.090 [1.045, 1.138]	<.001
Sex (female)	0.239 [0.068, 0.840]	.026
BMI (kg/m^2^)	0.515 [0.415, 0.641]	<.001
HGS (kg)	0.851 [0.780, 0.929]	<.001
GS (m/s)	0.015 [0.001, 0.206]	.002

*Note:* OR: odds ratio; CI: confidence interval; BMI: body mass index; HGS: handgrip strength; GS: gait speed.

^a^A *p* value less than .05 is statistically significant.

### Construction of a nomogram for predicting LSMI

**Figure 2.**
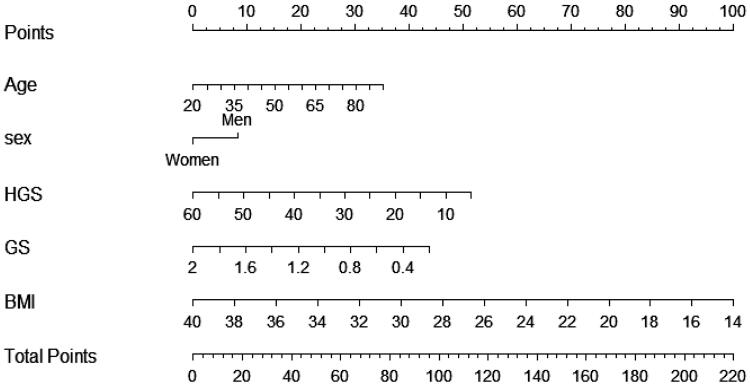
The nomogram for predicting LSMI based on the training cohort (*n* = 244). HGS: handgrip strength; GS: gait speed; BMI: body mass index.

The nomogram for LSMI was developed based on all the independent significant factors in the training set (Figure 2). Equations were extracted from the nomogram to calculate the total points for each patient. In the nomogram, the total points = 0.5025 × age (y) + 8.3022 × sex (male:1; female:0) − 0.9343 × HGS (kg) − 24.2520 × gait speed (m/s) − 3.8462 × BMI + 248.36.

### Discrimination and calibration

**Figure 3.**
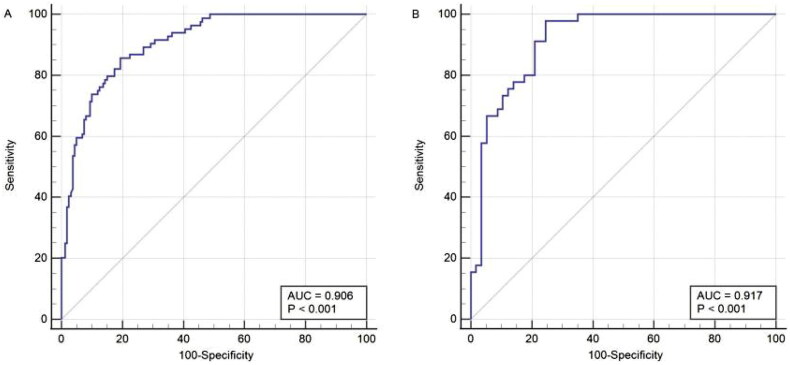
The ROC curves of nomogram for predicting LSMI in the training set (A, *n* = 244) and validation set (B, *n* = 102).

To examine the discriminative ability of the nomogram, the ROC curve of the nomogram was plotted, and the AUC was calculated (Figure 3). The AUC values for the LSMI nomogram were 0.906 (95% CI, 0.862–0.940) in the training cohort and 0.917 (95% CI, 0.846–0.962) in the validation cohort, indicating an excellent ability to discriminate between patients with and without LSMI.

In the training set, the optimal cutoff value of the total nomogram points was 157.3 for LSMI when the Youden index reached a maximum of 1.66, and the corresponding sensitivity and specificity were 85.71% and 80.62%, respectively. If a patient’s total score was higher than the cutoff threshold, he or she was identified as having LSMI. With this cutoff value, the sensitivity and specificity in the validation set were 80.00% and 80.70%, respectively. In the total population, the sensitivity and specificity were 83.72% and 80.65%, respectively (Table 4).

**Table 4.** Accuracy of the nomogram to predict low skeletal muscle mass index.

Variables	Total populations, *n* = 346	Training set, *n* = 244	Validation set, *n* = 102
Sensitivity (%)	83.72	85.71	80.00
Specificity (%)	80.65	80.62	80.70
Positive predictive value (%)	72.00	69.90	76.60
Negative predictive (%)	89.29	91.49	83.63
Consistency rate (%)	81.79	82.38	80.40

**Figure 4.**
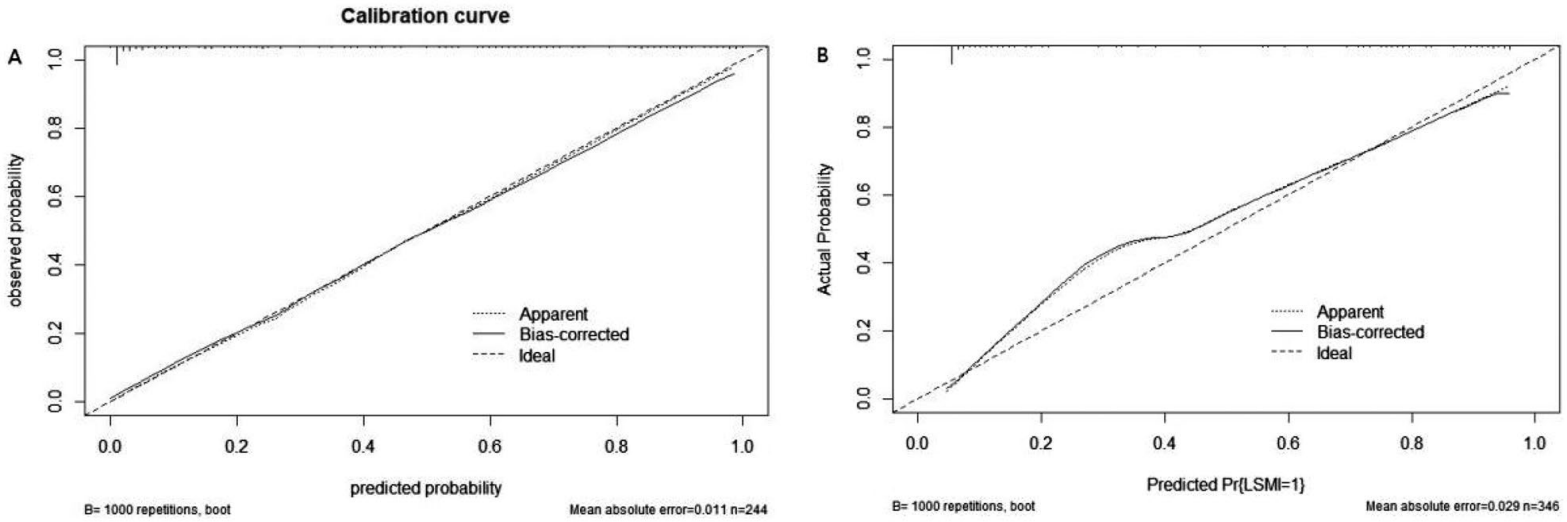
The calibration curves for predicting LSMI in the training (A, *n* = 244) and validation (B, *n* = 102) sets.

The calibration of the nomogram was checked by the calibration curve using the Hosmer–Lemeshow test. The calibration curves showed agreement between the observation and prediction (Figure 4), and the Hosmer–Lemeshow tests for the calibration analysis showed that the *p* values in the training and validation sets were .792 and .230, respectively, indicating that there was no deviation from the perfect fit.

### Clinical practicality

**Figure 5.**
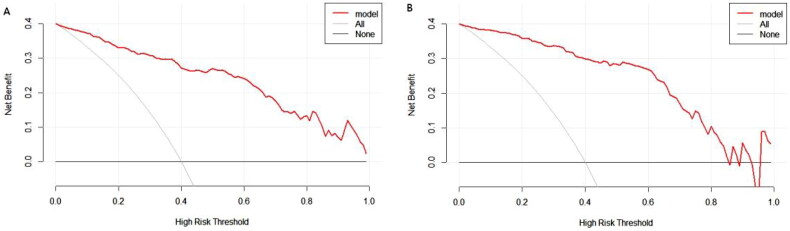
Decision curves for the proposed nomogram model in (A, *n* = 244) the training set and (B, *n* = 102) the validation set.

DCA was conducted for the prediction nomogram (Figure 5). The results indicate that utilizing the nomogram to predict LSMI may offer greater benefits than either treating all patients or treating none, for a threshold probability less than 0.85.

## Discussion

To the best of our knowledge, this is the first study to assess the predictive ability of easily available parameters, including BMI, SCr, HGS, and GS, for low muscle mass using a nomogram model. We established a model to predict LSMI. Age, sex, BMI, HGS, and GS were found to be independent predictive factors for LSMI. A diagnostic nomogram consisting of these factors successfully predicted LSMI with good accuracy and discrimination. Among the available methods for the assessment of muscle mass, we consider the nomogram model advantageous because LSMI can be easily assessed using this nomogram. The factors included in the nomogram model were practical and easy to measure.

Sarcopenia refers to the decline and dysfunction of skeletal muscle [27]. Protein energy wasting (PEW) is commonly used to describe the process of CKD-related wasting resulting from impaired kidney function [8]. It can be caused by compromised intake or assimulation of nutrients, as well as disease-associated inflammatory or other mechanisms [32]. Both sarcopenia and protein-energy wasting (PEW) are highly prevalent among patients undergoing HD [3,33]. Among these pathological conditions, low muscle mass is a prevalent component. The rapid method to diagnose low muscle mass may also provide clinicians with some diagnostic clues about these pathological conditions.

A recent study assessed the predictive ability of accessible factors for low muscle mass in patients with gastric cancer. Age, BMI, hemoglobin concentration, and GS were included in the nomogram model for predicting LSMI, and the diagnostic nomogram exhibited good discrimination, with an AUC of 0.818 in the training set and 0.809 in the validation set [12]. The factors included in the nomogram were similar to those used in our study. HGS was also included in the nomogram model in our study. Evidence suggests that the decline in muscle strength generally exceeds changes in muscle mass [34]. HGS has been recommended as a supportive measure of muscle mass using the GLIM criteria [9]. A recent study demonstrated that malnutrition, defined by GLIM criteria using HGS, could predict six-month mortality [11]. Second, HGS was reported to be closely correlated with other nutritional parameters, such as the protein index (assessed using neutron activation) [12]. Evidence suggests that HGS is a valid measure of nutritional status compared to malnutrition inflammation score (MIS) in patients on HD [35]. HGS is recommended as an indicator of protein energy status by KDOQI guidelines [28]. Third, HGS is reported to be associated with lean body mass (LBM) [16]. Fourth, it was demonstrated that HGS was improved with nutritional supplementation [36], indicating its use as a marker of nutritional interventions. These findings support its utility in predicting low muscle mass.

GS represents physical performance and is closely associated with lower extremity muscle mass [37]. It serves as a predictive indicator for mortality among patients undergoing HD [38], highlighting its significance in patient care. Physical performance not only predicts low muscle mass but also represents a potential therapeutic target. Aerobic exercise, for instance, may improve the physical performance and clinical outcomes of patients [39]. In our study, the combination of HGS and GS, along with age, sex, and BMI, performed well in predicting LSMI with an AUC of 0.906 in the training set and 0.917 in the validation set.

Aging is characterized by accelerated muscle loss [40]. Sex was also significantly associated with low muscle mass in our study. Males were at a higher risk of low muscle mass than females, which is consistent with a previous study [40,41]. Testosterone deficiency is a common finding among patients with end-stage renal disease (ESRD) [42]. Testosterone is an anabolic steroid that promotes skeletal muscle synthesis, and testosterone deficiency can easily lead to low muscle mass. BMI is the most commonly used surrogate of nutritional status. It was demonstrated that the higher BMI group had a greater LBM and fat mass (FM) [43]. An inverse relationship was observed between BMI and mortality [44].

We found that the association between serum albumin levels and low muscle mass disappeared in multivariate analysis. Although serum albumin is routinely tested in dialysis centers, it is not sufficiently sensitive to assess nutritional status. It may take months for sustained visceral protein depletion to develop hypoalbuminemia. It may also be influenced by other nonnutritional factors [28]. Serum creatinine was considered not only a uremic marker but also a predictor of nutritional status and muscle mass. However, it is affected by residual kidney function, dialysis adequacy, and dietary protein (meat) intake. In our study, serum creatinine levels were excluded from multivariate analysis. In addition to our research, Bataille et al. also did not recommend the use of serum creatinine levels in the prediction of low muscle mass [45]. The creatinine generation rate (CGR) derived from the creatinine kinetic model has also been proposed as an indicator of muscle mass. However, the relationship between CGR and muscle mass has been reported to be insufficiently strong and is affected by Kt/V [29].

For clinicians, a cost-effective and simplified method for the rapid diagnosis of low muscle mass is highly desirable. Among the variables that were incorporated into the nomogram model in our study, GS is widely used in practice due to its rapidity, security, and high reliability [15]. Similarly, HGS is also recommended for regular use in both hospital settings and community healthcare practices [46,47]. In addition, body weight measurements were taken after each dialysis session for HD patients. Therefore, we believe that the nomogram model is beneficial not only for regular evaluation but also for routine monitoring among patients undergoing HD.

This study has some potential limitations. First, we used BIA, rather than DEXA, to diagnose LSMI. The accuracy of muscle mass measurement by BIA in hemodialysis patients has been confirmed and applied in multiple studies [29,45,48–53], and it was recommended to assess body composition according to the KDOQI guidelines [28]. To eliminate the effect of excess fluid, we excluded patients with severe edema, and the time of the measurements was set after the end of HD, when they approached the ideal weight. Thus, we considered the accuracy of muscle mass assessment using BIA to be reliable. Second, we excluded patients with the highest risk of LSMI, such as patients confined to bed or a wheelchair. Therefore, further studies are needed before extrapolating these results to the aforementioned excluded patients. Finally, although our findings were validated in the validation cohort, the sample size was relatively small, and studies with a larger number of patients are needed to further verify the reliability of our results.

## Conclusion

We first developed a prediction model to identify LSMI in patients undergoing HD. The nomogram model included age, sex, BMI, HGS, and GS. It successfully predicted the presence of LSMI. This nomogram provides an accurate visual tool for early identification and timely intervention.
